# Wettability of Y_2_O_3_: A Relative Analysis of Thermally Oxidized, Reactively Sputtered and Template Assisted Nanostructured Coatings

**DOI:** 10.3390/nano2010065

**Published:** 2012-02-29

**Authors:** Harish C. Barshilia, Archana Chaudhary, Praveen Kumar, Natarajan T. Manikandanath

**Affiliations:** 1 Surface Engineering Division, CSIR-National Aerospace Laboratories, Bangalore-560017, India; Email: harish@nal.res.in (H.C.B); Email: archana@nal.res.in (A.C); Email: kumar@nal.res.in (P.K); Email: manint@nal.res.in (N.T.M)

**Keywords:** yttrium oxide, cadmium oxide, sputtering, template assisted growth, wettability, work of adhesion, thermal oxidation, surface roughness

## Abstract

The wettability of reactively sputtered Y_2_O_3_, thermally oxidized Y-Y_2_O_3_ and Cd-CdO template assisted Y_2_O_3 _coatings has been studied. The wettability of as-deposited Y_2_O_3 _coatings was determined by contact angle measurements. The water contact angles for reactively sputtered, thermally oxidized and template assisted Y_2_O_3 _nanostructured coatings were 99°, 117° and 155°, respectively. The average surface roughness values of reactively sputtered, thermally oxidized and template assisted Y_2_O_3 _coatings were determined by using atomic force microscopy and the corresponding values were 3, 11 and 180 nm, respectively. The low contact angle of the sputter deposited Y_2_O_3_ and thermally oxidized Y-Y_2_O_3_ coatings is attributed to a densely packed nano-grain like microstructure without any void space, leading to low surface roughness. A water droplet on such surfaces is mostly in contact with a solid surface relative to a void space, leading to a hydrophobic surface (low contact angle). Surface roughness is a crucial factor for the fabrication of a superhydrophobic surface. For Y_2_O_3_ coatings, the surface roughness was improved by depositing a thin film of Y_2_O_3 _on the Cd-CdO template (average roughness = 178 nm), which resulted in a contact angle greater than 150°. The work of adhesion of water was very high for the reactively sputtered Y_2_O_3_ (54 mJ/m^2^) and thermally oxidized Y-Y_2_O_3_ coatings (43 mJ/m^2^) compared to the Cd-CdO template assisted Y_2_O_3_ coating (7 mJ/m^2^).

## 1. Introduction

The wettability property of a solid surface is important scientifically and technologically. The surface morphological structure and surface chemistry mainly control the wetting or repellant behavior of the solid surface. Contact angle measurement commonly determines the wettability of a solid surface. For a liquid on a flat solid surface the contact angle is measured as the combined result of three different types of interface tension at the solid, liquid and gas interfaces given by the classical Young’s equation:


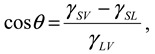
(1)

where *γ*_SV_, *γ*_SL_, and *γ*_LV_ are the interfacial free energies per unit area of solid-vapor, solid-liquid, and liquid-vapor interfaces. Young's equation tells us that hydrophobicity refers to a contact angle greater than 90° while a contact angle less than 90° implies hydrophilicity. Solid surfaces are usually not perfectly flat but are somewhat rough, so the effect of surface roughness has to be considered for surface wettability. The Wenzel and Cassie-Baxter models describe the effect of morphological parameters such as surface roughness on the wettability of solid surfaces [1,2]. The Wenzel model assumes that the liquid can enter completely and contact the concave regions on the solid surface. Young’s equation was modified by Wenzel by including a roughness factor and the modified equation is:


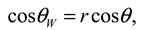
(2)

where *θ_W_* is the apparent contact angle on a rough surface and *r* is the ratio of the actual to the projected area. If air can be trapped by the liquid to give a composite surface, the latter belongs to Cassie’s case and the apparent contact angle is described as the modified equation:


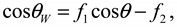
(3)

where, *f_1_* and *f_2_* are the fractional interfacial areas of solid and air trapped between the solid surface and the water droplet, respectively and 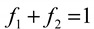. This model clearly demonstrates that the larger the vapor fraction (*f*_2_) the more hydrophobic is the surface.

A great deal of attention is now being placed on inorganic nanomaterials because of their various potential applications in the production of electronic devices, sensors, biochips and energy storage media [3,4,5,6,7,8,9,10,11,12]. Inorganic materials have also been used to form superhydrophobic surfaces [13,14,15]. Recently, with the development of smart devices, such as the intelligent microfluidic switch [16], reversibly controlling the surface wettability has aroused great interest and has been realized by using external stimuli such as heating/cooling, light irradiation and temperature [17,18,19]. Several stimuli-responsive, smart, interfacial materials that can be switched between superhydrophilicity and superhydrophobicity by combining the geometrical morphology of the surface with a change of surface chemistry have been reported [20,21,22]. With a high dielectric constant (10–17), high melting point (2439 °C), high refractive index (1.7–1.9) and large optical band gap (5.5 eV) yttrium oxide (Y_2_O_3_) is a very promising material for the potential applications mentioned above [23,24]. Combining the properties listed above with superhydrophobicity opens up new possibilities for the use of Y_2_O_3_ in diverse fields. The ability of yttrium oxide to be a host material for the rare earth atoms europium or thulium makes it an important material for optical applications [25,26,27,28,29]. The Eu^3+^ doped Y_2_O_3_ is a well-known red phosphor [25]. The Y_2_O_3_ thin films have been deposited by several deposition techniques: radio frequency (RF) magnetron sputtering, pulsed laser ablation, ion-beam sputtering, solvothermal process, hydrothermal reaction, wet-chemical method, physical vapor deposition (PVD) methods and reactive sputtering [26,30,31,32,33,34,35,36]. Yttrium oxide through synthesis has enabled the creation of structures such as nanoparticles, nanotubes, nanorods, nanospheres, nanoflowers, *etc*. [25,37,38]. To the best of our knowledge superhydrophobicity for yttrium oxide based coatings has not been reported so far.

In this paper three different types of yttrium oxide based coatings: sputter deposited Y_2_O_3_, thermally oxidized Y-Y_2_O_3_ and template assisted Y_2_O_3_ were prepared and the effect of surface morphology on the wettability was studied. A contact angle goniometer was used to investigate the static contact angle (CA) of the coatings. The structural and chemical properties of these coatings have been studied using X-ray diffraction (XRD), atomic force microscopy (AFM), micro-Raman spectroscopy and field emission scanning electron microscopy (FESEM).

## 2. Experimental Details

The Y_2_O_3_ coatings were prepared on borosilicate glass substrates by using three different approaches. The first set of Y_2_O_3_ coatings were deposited by sputtering a high purity (99.99%) yttrium target (0.076 m diameter and 0.006 m thickness) in Ar + O_2_ plasma using an RF generator (*f *= 13.56 MHz, hereafter called as Sample 1). The sputtering process parameters were optimized by preparing Y_2_O_3_ coatings at different power levels, substrate temperatures and O_2_ flow rates. The optimized process parameters were: Ar flow rate = 25 sccm (standard cubic centimeter per minute), O_2_ flow rate = 2 sccm, target power = 350 W and duration = 60 min. The substrates were chemically cleaned in an ultrasonic agitator by isopropyl alcohol and acetone before placing them in the vacuum chamber. The vacuum chamber was pumped down to a base pressure of 5.0 × 10^–4^ Pa. The second set of Y_2_O_3_ coatings was prepared by thermal oxidation of sputter deposited yttrium. The Y-Y_2_O_3 _coatings were prepared by depositing yttrium using 85 W of direct current (DC) power for 11 min followed by oxidation (O_2_ flow rate = 75 sccm) at 350 °C for 2 h. Hereafter, this coating is referred to as Sample 2. In the third set, coatings were prepared by depositing Y_2_O_3_ on the Cd-CdO template. The preparation of the Cd-CdO template is reported elsewhere [39]. In brief, the cadmium coating was deposited using 85 W of RF power for 4 min and subsequently oxidized in an oxygen atmosphere (O_2 _flow rate = 85 sccm) at 225 °C for 2 h, thus forming a Cd-CdO template. The Cd-CdO coatings prepared under these conditions were superhydrophobic in nature [39]. The thin films of Y_2_O_3_ were deposited on a Cd-CdO template by the reactive sputtering technique. Y_2_O_3 _thin films were deposited under the following optimized conditions: Ar flow rate = 25 sccm, O_2_ flow rate = 2 sccm, target power = 350 W and duration = 10 min. The thickness of the Y_2_O_3 _film on the glass substrate was found to be about 60 nm for a 10 min deposition at 350 °C (hereafter called Sample 3). The Y_2_O_3_ samples were prepared with a thickness of 10–80 nm on Cd-CdO templates.

The static contact angle was measured according to the sessile-drop method using a contact angle analyzer (Phoenix 300 Goniometer, Surface Electro Optics Co., Suwan City, Gyunggido, Korea) with three different liquids (water, formamide and glycerol). The system consists mainly of a CCD video camera with a resolution of 768 × 576 pixels. The drop image was stored by a video camera and an image analysis system was used to calculate the left and right angles from the shape of the drop with an accuracy of ±0.1°. The droplet size of the fluid was about 5 µL, therefore, the gravitational effect can be neglected. The contact angle of the samples was measured at three different places and the values reported herein are the average of three measurements. The dynamic contact angle measurements were also carried out using a Rame-Hart contact angle goniometer (model 100-00) equipped with a CCD camera. For these measurements, we took a 8 micro-liter droplet on the substrate and then again added 4 micro-liter of water to the same droplet.

The chemical structure of the coatings was studied using micro-Raman spectroscopy. A DILOR-Jobin-Yvon-SPEX integrated micro-Raman spectrometer was used for the present study. Three-dimensional surface imaging of the coatings was measured by atomic force microscopy (Surface Imaging System) in the contact mode. A Si based tip (Nanosensors) with a radius of less than 5 nm was used for the AFM measurements. The microstructure of the coatings was studied using field emission scanning electron microscopy (Supra 40VP, Carl Zeiss, Oberkochen, Germany). The X-ray diffraction patterns of the coatings were recorded in a X-ray diffractometer system (JEOL, JDX-8030) with thin film attachment (α = 3°). The X-ray source was CuK_α_ radiation (λ = 0.15418 nm), which was operated at 35 kV and 20 mA.

## 3. Results and Discussion

### 3.1. Structure and Morphology

#### 3.1.1. X-ray Diffraction

**Figure 1:**
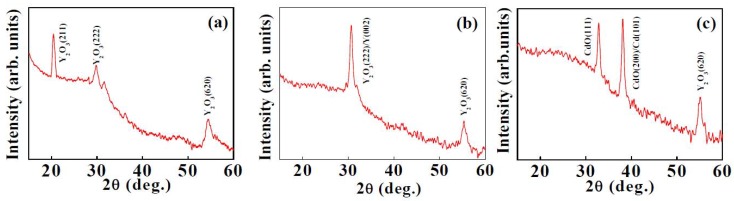
X-ray diffraction plots of: (a) sputter deposited Y_2_O_3 _coating, (b) thermally oxidized Y-Y_2_O_3_coating, and (c) template assisted Y_2_O_3 _coating.

Figure 1(a–c) shows the XRD plots of sputter deposited Y_2_O_3_, thermally oxidized Y-Y_2_O_3_ and Cd-CdO template assisted Y_2_O_3_ coatings. Reactively sputtered coatings (Figure 1(a)) showed reflections corresponding to (211), (222) and (620) of cubic Y_2_O_3_ (JCPDS card no. 025–1200). Figure 1(b) shows XRD pattern for thermally oxidized Y-Y_2_O_3_ coating. Two peaks are observed at 2θ = 30.7° and 55.3°, which correspond to (222) and (620) planes of cubic Y_2_O_3_,respectively (JCPDS card no. 025–1200). The peak centered at 2θ = 30.7° corresponds to (002) plane of hexagonal yttrium (JCPDS card no. 12–702). The presence of diffraction peaks from Y and Y_2_O_3 _show that only the top surface layer of the coating is oxidized. The XRD plot for Cd-CdO template assisted Y_2_O_3 _coating is shown in Figure 1(c). The peak observed at 2θ = 55.3° corresponds to the (620) plane of Y_2_O_3_. Two additional peaks are also observed at 2θ = 32.9° and 38.1°, which are attributed to the (111) and (200) planes of cubic CdO, respectively (JCPDS card no. 5–0640). The peak centered at 2θ = 38.1° corresponds to (101) plane of metallic hexagonal Cd (JCPDS card no. 5–0674). The diffraction peaks for Cd and CdO are from the template. Here we observed diffraction peaks for both Cd and CdO, which indicates that only the uppermost surface layer of the coating is oxidized, as the Cd-CdO template is prepared by sputtering followed by thermal oxidation. The average grain size of Y_2_O_3 _coatings was calculated from the prominent peaks using Scherrer’s formula. The average grain sizes for sputtered deposited Y_2_O_3_, thermally oxidized Y-Y_2_O_3_ and template assisted Y_2_O_3_ coatings were 15.0 nm, 15.5 nm and 19.5 nm, respectively.

#### 3.1.2. Micro-Raman Spectroscopy

**Figure 2:**
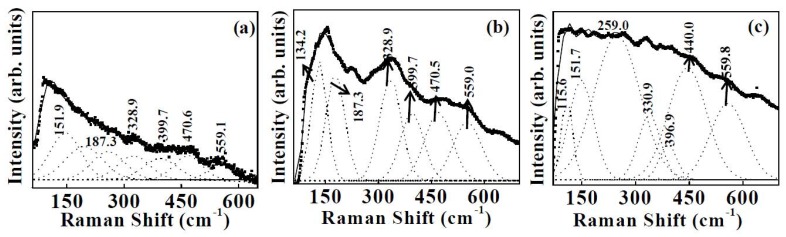
Deconvoluted Raman spectra of: (a) sputter deposited Y_2_O_3 _coating, (b) thermally oxidized Y-Y_2_O_3 _coating, and (c) template assisted Y_2_O_3 _coating.

The chemical structure of the coating was studied by micro-Raman spectroscopy (Figure 2). Yttrium sesquioxide crystallizes in the cubic system and is a body centered cubic with space group Ia3 (Z = 16). The structure is related to the structure of fluorite, with each yttrium ion located at the center of the cube from which two of the eight neighboring oxygens of the fluoride have been removed. As the structure is body centered, the unit cell contains the primitive cell twice. The latter cells, containing eight formula units, were used for the theoretical numbering of vibration. The irreducible representations for optical and acoustical modes are [40,41]:

Г_op_ = 4 A_g _+ 4 E_g_+ 14 F_g_+ 5 A_2u _+ 5 E_u_+ 16 F_u_

Г_ac_ = Fu,

where A_g_, E_g_ and F_g_ are Raman active, F_u_ is infrared (IR) active and A_2u_ and E_u_ are inactive. Twenty two Raman lines of A_g_, E_g_ and F_g_ modes and sixteen F_u_ IR bonds are then predicted. Figure 2(a–c) shows the Raman spectra of Samples 1–3. The frequencies of various Raman bands were determined using deconvolution of the Raman data by Gaussian fit as shown in Figure 2. The assignment of the Raman spectrum of cubic Y_2_O_3_ for Samples 1–3 is given in Table 1. The Raman spectrum of Sample 1 (Figure 2(a)) showed six peaks at 151.9, 187.3, 328.9, 399.7, 470.6 and 559.1 cm^−1 ^[40,41]. The peaks observed at 187.3, 328.9 and 559.1 cm^−1^ are attributed to the F_g_+ E_g_ mode of Y_2_O_3 _[40,41]. The peak centered at 399.7 cm^−1^ corresponds to F_g_ and at 151.9, 470.6 cm^−1^ correspond to F_g_ + A_g_ modes of Y_2_O_3_[40,41]. For Sample 2, the Raman peaks observed at 134.2 and 399.7 cm^−1^ are attributed to F_g_, 187.3, 328.9 and 559.0 cm^−1 ^are attributed to F_g_ + E_g_, and 470.6 cm^−1 ^is attributed to F_g_ + A_g _modes of cubic Y_2_O_3_ (Figure 2(b)) [40,41]. The Raman spectrum of template assisted Y_2_O_3 _coating (Sample 3) is shown in Figure 2(c). In addition to the above modes we observed a few additional peaks at 115.6, 440.0 and 559.8 cm^−1^. The peaks centered at 115.6 and 396.9 cm^−1^ are attributed to F_g_ + A_g _and F_g_ modes of Y_2_O_3_, respectively. The peaks observed at 440.0 and 559.8 cm^−1^ correspond to F_g_+ E_g_ mode of Y_2_O_3 _[40,41]. For Sample 3 an additional peak is observed at 259.0 cm^−1^, which corresponds to CdO. This peak is from the Cd-CdO template.

**Table 1:** The assignment of Raman spectra of for reactively sputtered Y_2_O_3_, thermally oxidized Y-Y_2_O_3_ and template assisted Y_2_O_3 _coatings.

Sample 1	Sample 2	Sample 3
Peak position (cm^−1^)	Symmetry	Peak position (cm^−1^)	Symmetry	Peak position (cm^−1^)	Symmetry
151.9	F_g_+ A_g_	134.2	F_g_	115.6	F_g_
187.3	F_g_+ E_g_	187.3	F_g_+ E_g_	151.7	F_g_+ A_g_
328.9	F_g_+ E_g_	328.9	F_g_+ E_g_	330.9	F_g_+ E_g_
399.7	F_g_	399.7	F_g_	396.9	F_g_
470.6	F_g_+ A_g_	470.5	F_g_+ A_g_	440.0	F_g_+ E_g_
559.1	F_g_+ E_g_	559.0	F_g_+ E_g_	559.8	F_g_+ E_g_

#### 3.1.3. Wettability of Y_2_O_3 _Coatings

**Figure 3:**
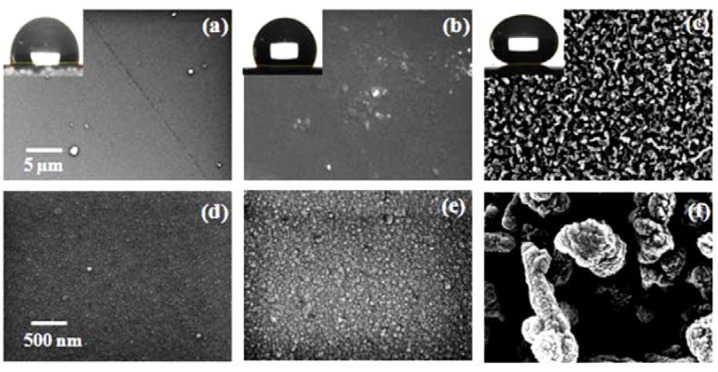
Field emission scanning electron microscopy images of: (a) sputter deposited Y_2_O_3 _coating, (b) thermally oxidized Y-Y_2_O_3 _coating, and (c) template assisted Y_2_O_3 _coating at low magnification with the corresponding optical photographs of water droplet contact angle shown in the inset. High magnification images are shown in (d–f), respectively.

Sputter deposited Y_2_O_3_, thermally oxidized Y-Y_2_O_3_ and template assisted Y_2_O_3 _coatings show different surface morphologies. The surface morphologies of Samples 1–3 were examined by FESEM and are shown in Figure 3(a–f) at two different magnifications. The water contact angles for Samples 1, 2 and 3 were 99°, 117° and 155°, respectively as shown in the insets in Figure 3(a–c). The dynamic contact angle measurements for Sample 3 showed an advancing water contact angle of 154° and a receding water contact angle of 144° with a contact angle hysteresis of 8° (data not shown). The relatively low contact angle of the sputter deposited Y_2_O_3_ and thermally oxidized Y-Y_2_O_3_ coatings compared to the template assisted Y_2_O_3 _coating is attributed to a densely packed nano-grain like microstructure without any void space (shown in Figure 3(a,b) and Figure 3(d,e) at lower and higher magnifications, respectively). The sputter deposited and thermally oxidized Y_2_O_3 _coatings show hydrophobicity with an ultralow surface roughness (discussed later). It is well known that, improving the surface roughness is a crucial factor for the fabrication of a superhydrophobic surface [42]. To improve the surface roughness a new process was applied. The Cd-CdO template was prepared with a high average surface roughness (R_a_ = approximately 178 nm, data not presented) and shows superhydrophobic nature (water contact angle > 150°). A thin film of Y_2_O_3 _is deposited on the Cd-CdO template which enhanced the surface roughness, resulting in the superhydrophobicity. The surface morphology of the template assisted Y_2_O_3 _coating is shown in Figure 3(c,f) at low and high magnifications, respectively.

**Figure 4:**
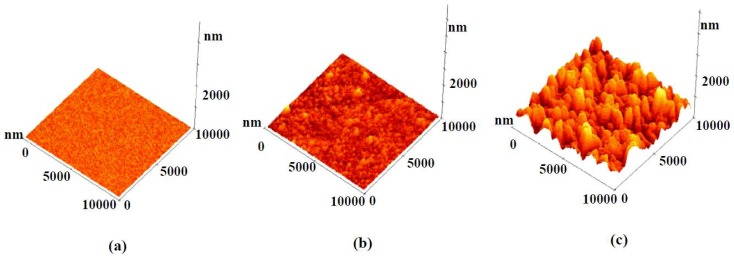
3D atomic force microscopy (AFM) images of: (a) sputter deposited Y_2_O_3 _coating, (b) thermally oxidized Y-Y_2_O_3 _coating, and (c) template assisted Y_2_O_3 _coating.

**Figure 5:**
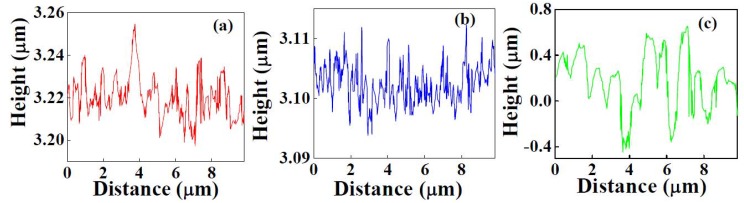
Surface roughness profiles of: (a) sputter deposited Y_2_O_3 _coating, (b) thermally oxidized Y-Y_2_O_3 _coating, and (c) template assisted Y_2_O_3 _coating.

**Figure 6:**
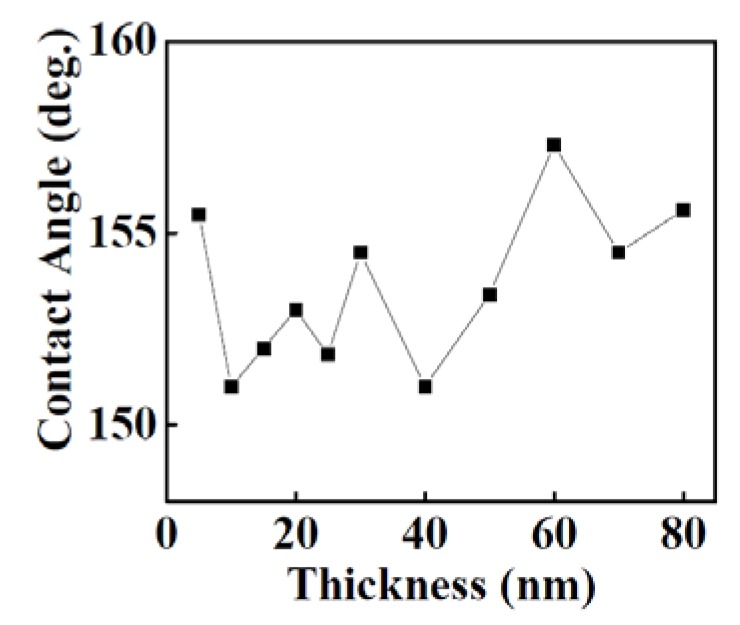
Variation of contact angle of Cd-CdO template assisted Y_2_O_3_ coating with thickness of Y_2_O_3 _layer.

**Figure 7:**
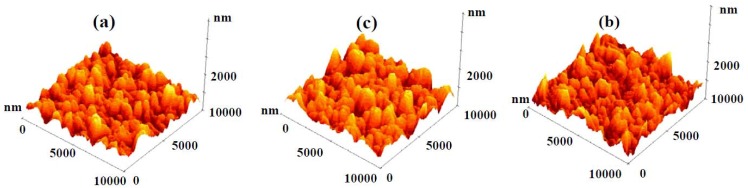
AFM images of template assisted Y_2_O_3 _coating at different thicknesses of Y_2_O_3_ layer: (a) 30 nm, (b) 60 nm, and (c) 80 nm.

In order to further confirm the microstructure of these samples the FESEM data was also recorded at higher magnification, which showed a nanograin-like microstructure for sputtered deposited Y_2_O_3_ (Figure 3(d)), thermally oxidized Y-Y_2_O_3 _(Figure 3(e)) and non-uniform nanostructures for the template assisted Y_2_O_3_ coating (Figure 3(f)). The low resolution image (Figure 3(c)) shows the presence of solid components (*i.e.*, whitish regions) and air pockets (*i.e.*, darker regions). This combination of air gaps and solid regions behaves as the first or higher scale roughness of the coating as shown in the roughness profile presented later. Even though the air pockets are in sub-micron range, the roughness (that is, average height of hills and valleys) was in the nanometric scale. The high magnification (Figure 3(f)) showed the solid surface to consist of fused individual structures which generated a textured or a patterned surface. The textured surface acts as the second or lower scale roughness (believed to be a few tens of nanometers). This texturing gives rise to a multi-scale roughness in the Y_2_O_3_ coating deposited on the Cd-CdO template, which was responsible for the observed superhydrophobicity. According to the Cassie-Baxter model, the surface fraction of the solid (*f*_1_) and air pockets (*f*_2_) impacts the water contact angle for a composite surface which can be calculated by Equation 3. The air fractions for Samples 2 and 3 were 0.32 and 0.91, respectively (assuming Sample 1 with an average roughness 3 nm as a smooth surface and contact angle for Sample 1 = 99°). The surface roughness of as-deposited Y_2_O_3_ coatings was measured by AFM. Figure 4(a–c) shows the AFM images of sputter deposited Y_2_O_3_, thermally oxidized Y-Y_2_O_3_ coating and template assisted Y_2_O_3_ coating and the corresponding 2D roughness profiles are shown in Figure 5(a–c). The values of average surface roughness of sputter deposited Y_2_O_3_, thermally oxidized Y_2_O_3 _and template assisted Y_2_O_3_ coatings are: 3, 11 and 180 nm, respectively. The AFM data confirms the presence of nanoscale surface roughness. The presence of air-pockets in Sample 3 was confirmed by FESEM as shown in Figure 3(c), which contributes to the higher scale of roughness. The variation of the water contact angle with the template assisted Y_2_O_3_ coating thickness is shown in Figure 6. It is clearly seen that irrespective of the change in the thickness of the coating, the contact angle always remains greater than 150°. To cross check this observation, the surface roughness of the coatings was measured by AFM. The AFM images of the template assisted Y_2_O_3_ coating for different thicknesses are shown in Figure 7(a–c). The average surface roughness values for 30, 60 and 80 nm thickness of template assisted Y_2_O_3_ coatings are approximately 180, 185 and 188 nm, respectively. The Y_2_O_3_ coating follows the template/substrate morphology. It can be concluded that for coatings with different thicknesses of Y_2_O_3_, the change in the average surface roughness was negligible, which is responsible for superhydrophobicity for all thicknesses.

**Figure 8:**
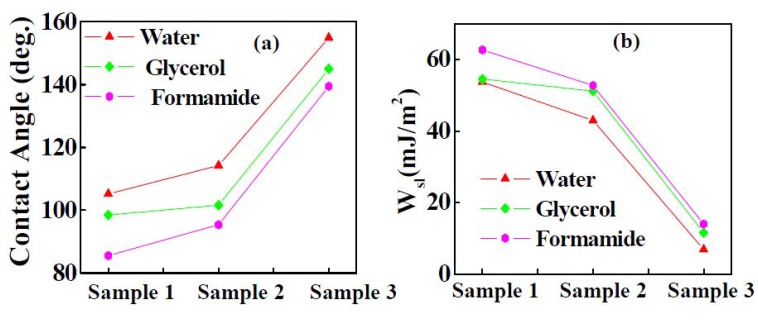
Variations of: (a) contact angle and (b) work of adhesion of the polar liquids for Samples 1–3.

The contact angle and work of adhesion of the polar liquids for Samples 1–3 are shown in Figure 8(a,b), respectively as per the procedure described elsewhere [43]. Ideally the static CAs of the sample should be found experimentally using a series of probe liquids, with a balanced composition of polar and non-polar components. A suggested series of probe liquids that are easily available and cover the whole range of surface tension values are, polar protic–water, glycerol, formamide and aniline, and non-polar aprotic–diiodomethane, dodecane, hexadecane, ethylene glycol and benzene. In the present work we chose only three liquids for the initial estimate of the free surface energy of the samples. The dispersive and polar components of glycerol, formamide and water are reported in Reference 44. The static contact angles for Samples 1–3 for different probe liquids are presented in Table 2. Compared to non-polar liquids, the polar liquids interact differently with Y_2_O_3 _coatings because they exhibit a large dipole moment and have a strong tendency for hydrogen bonding. The contact angle of liquids (water, formamide and glycerol) for Samples 1–3 increased in the following order: *θ*_formamide_θ_glycerol_θ_water_. The free energies of the liquid-solid, solid-vapor and liquid-vapor interfaces are dependent on the work of adhesion at the solid-liquid interface. The work of adhesion for Samples 1–3 is calculated, without taking into account the polar/apolar interactions by the Young-Dupre Equation [45]:

(4)

The work of adhesion of water for Samples 1–3 were 54, 43 and 7 mJ/m^2^, respectively. It can be clearly seen that the work of adhesion for Sample 3 was very low compared to Samples 1 and 2. This is because the template assisted Y_2_O_3 _coating demonstrated a composite surface (Cassie Baxter state), which contains a void space filled with air combined with nonuniform nanostructures (shown in Figure 3(c)). This composite interface decreases the solid-liquid contact area and therefore decreases the work of adhesion. These results show that the presence of a micron scale void space combined with non-uniform nanostructures like morphology is responsible for superhydrophobicity in Sample 3.

**Table 2:** Static contact angles of the probe liquids for reactively sputtered Y_2_O_3_, thermally oxidized Y-Y_2_O_3_ and Cd-CdO template assisted Y_2_O_3_ coatings.

Sample	Static contact angle (degree)
Water	Glycerol	Formamide
Sample 1	99	98	85
Sample 2	117	101	95
Sample 3	155	145	139

## 4. Conclusions

The water contact angles were 99°, 117° and 155° respectively for reactively sputtered Y_2_O_3_, thermally oxidized Y-Y_2_O_3_ and the Cd-CdO template assisted Y_2_O_3_ coating. Superhydrophobicity was demonstrated by the template assisted Y_2_O_3 _coating and it was attributed to an optimum combination of non-uniform nanostructures and a void space which gives rise to a high surface roughness. The work of adhesion was calculated for different probe liquids (water, glycerol and formamide). The work of adhesion for all three liquids was very low for the Cd-CdO template assisted Y_2_O_3 _coating when compared to the reactively sputtered Y_2_O_3 _coating and the thermally oxidized Y-Y_2_O_3 _coating. The superhydrophobic properties of the the Cd-CdO template assisted Y_2_O_3 _coating are attributed to a non-uniform nanostructure like morphology combined with a micron scale void space filled with air. The water droplet on such a coating is in contact with a comparatively higher fraction of air gaps, than the rough surface which results in a higher contact angle. The surface roughness of reactively sputtered Y_2_O_3 _(3 nm), thermally oxidized Y-Y_2_O_3_ (11 nm) and the Cd-CdO template assisted Y_2_O_3_ coating (180 nm) was determined by AFM. The reactively sputtered Y_2_O_3 _and thermally oxidized Y-Y_2_O_3 _coatings demonstrate hydrophobicity with a very low surface roughness. The surface roughness of the Y_2_O_3 _coating was improved by using a Cd-CdO template, which resulted in superhydrophobicity.
